# A rare case of digital artery aneurysm

**DOI:** 10.1016/j.jpra.2019.03.002

**Published:** 2019-03-19

**Authors:** Zak Vinnicombe, Max Little, Alexi Nicola, Javier Ibañez

**Affiliations:** a Department of Plastic Surgery, St. Thomas’ Hospital, Westminster Bridge Road, London SE1 7EH, United Kingdom; b Department of Trauma and Orthopaedics, St. Mary's Hospital, Praed Street, London W2 1NY, United Kingdom

**Keywords:** Digital artery aneurysm, Plastic surgery, Hand surgery, Microvascular surgery

## Abstract

True aneurysms of the arteries of the hand are vanishingly rare. We report a case detailing the surgical management of a 44-year old gentleman with a true common digital artery aneurysm. This report adds to the current sparse literature on digital artery aneurysms and their presentation, investigation and management.

## Introduction

An aneurysm is defined as a permanent, localised dilatation of an artery to >150% of its expected size. True aneurysms of the arteries of the hand are vanishingly rare. A 2017 literature review yielded only 23 cases of true digital artery aneurysms with the vast majority of these cases being secondary to trauma.^1^ It has been suggested that the mechanism of true aneurysms is repeated blunt or vibrational trauma to the area.^2^ In contrast, false aneurysms are reported to occur after penetrating trauma. Other aetiologies include infection, atherosclerosis, inflammatory and congenital causes. In this article we describe the presentation, investigation and surgical management of a patient with a true common digital artery aneurysm.

## Case report

A 44-year-old gentleman was referred to the vascular surgery team by his GP with a several month history of a lump on the volar aspect of his right hand. He did not recall any significant or repetitive trauma. His past medical history includes only well-controlled asthma and primary Raynaud Syndrome. Of note, he is a musician and has played multiple instruments, including saxophone, clarinet, piano and guitar, for most of his life. He is also a regular golfer. He has no family history of aneurysms and is a lifelong non-smoker.

**Figure 1:**
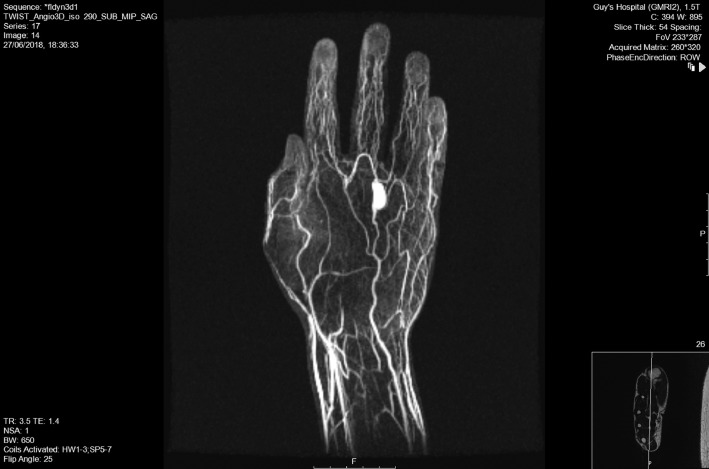
Magnetic resonance angiography showing common digital artery aneurysm.

An initial ultrasound scan showed a pulsatile, anechoic sac measuring 18.9 × 9.3 mm in size, consistent with an aneurysm. A subsequent magnetic resonance angiogram (MRA) confirmed a 16 × 7 mm saccular aneurysm of the common digital artery between the middle and ring fingers (Figure 1). The dynamic contrast study showed good collateral supply to the right middle and ring fingers. The patient was referred to the plastic surgery hand team due to concerns with the potential complexity of surgical management.

After discussion with the team, the patient opted to proceed with surgical excision and understood that a vein graft may be required if blood supply to the fingers was affected by removal of the aneurysm.

**Figure 2:**
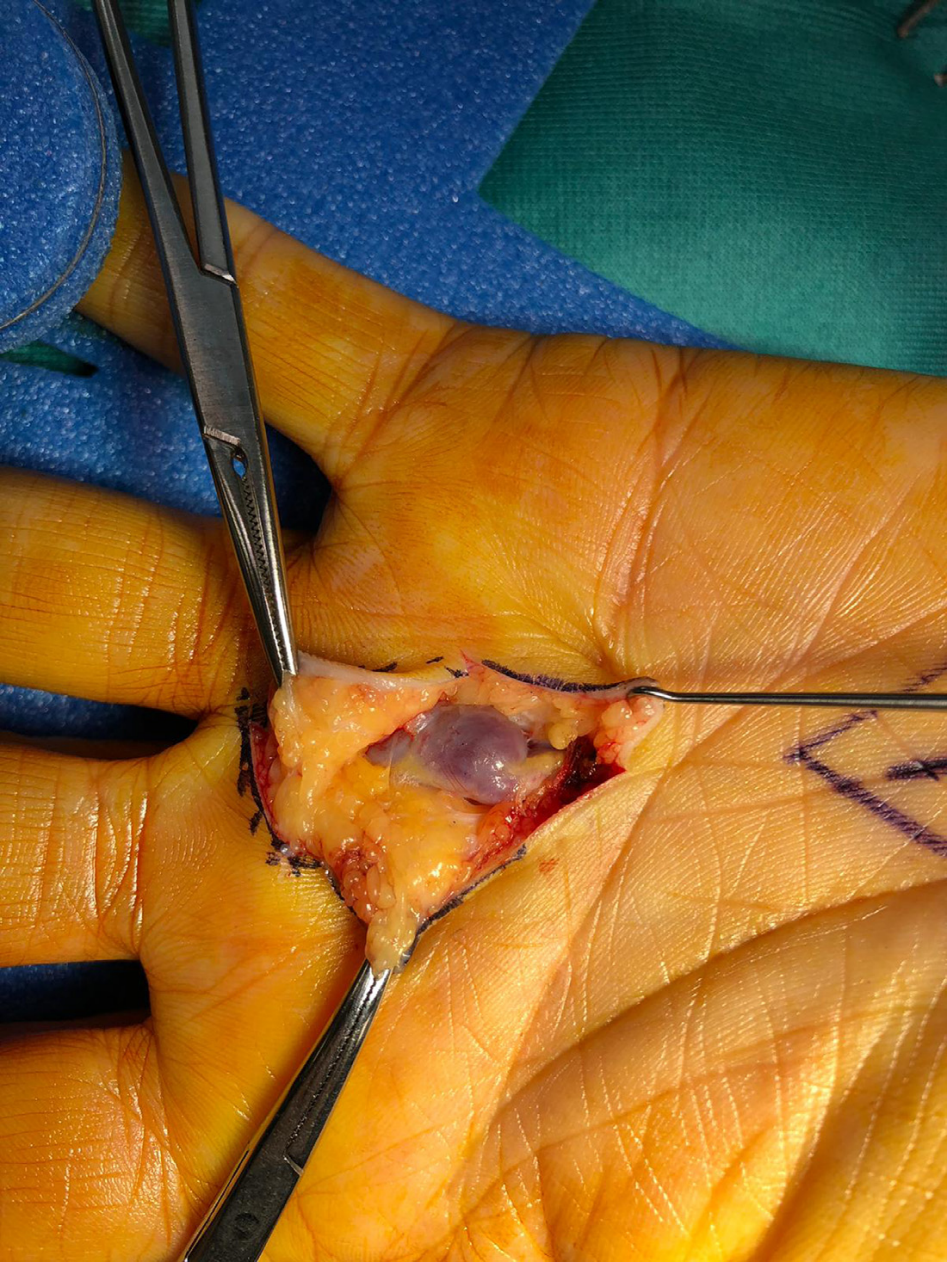
Aneurysm intraoperatively, arising from common digital artery supplying the radial side of the right ring finger and ulnar side of the right middle finger.

**Figure 3:**
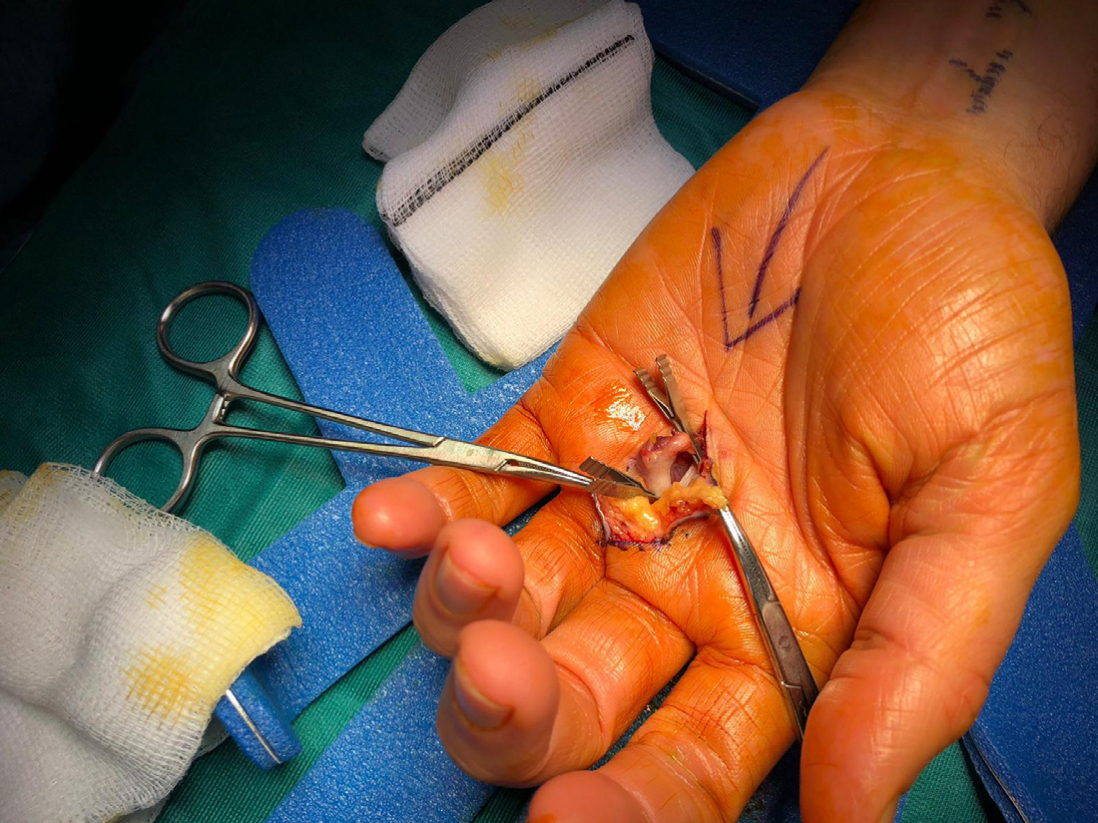
Microvascular clamps proximal and distal to aneurysm.

Preoperative modified Allen test was normal and the patient underwent excision of the lesion as a day case procedure under a general anaesthetic with brachial plexus block supplementation and arm tourniquet. The lesion was confirmed intraoperatively as a true aneurysm of the common digital artery supplying the radial side of the right ring finger and ulnar side of the right middle finger (Figure 2). The aneurysm was clamped both proximally and distally and good distal perfusion was confirmed (Figure 3). The aneurysm was excised and sent for histology, both ends were tied off and the skin closed. Perfusion of the fingers was confirmed after application of dressings. Histology confirmed the diagnosis of true aneurysm. At one-month follow-up the patient had no ongoing sequelae.

## Discussion

Due to the paucity of literature on digital artery aneurysms, aetiological evidence is lacking. ‘Hypothenar hammer syndrome’ is a well-described, similar condition with evidence demonstrating the role of chronic, repetitive trauma.^2^ However, as there are so few documented cases of digital artery aneurysms, causation has not yet been established.

Previous case reports have suggested that mechanisms of chronic trauma causing digital artery aneurysm include bowling, volleyball, baseball, golf and repetitive trauma from a wedding ring.^3^ Interestingly, a previous case describing a ‘spontaneous’ aneurysm in a clarinet player has been reported.^4^

Bouvet and colleagues have recently suggested an imaging algorithm for diagnosis of aneurysms of the hand.^5^ Ultrasound scanning is the primary modality for diagnosis of palpable masses in the hand which can then be supplemented by MRA if there is no evidence of acute ischaemia. This was the approach used for diagnosis in our case and serves to further strengthen the evidence for MRA as a diagnostic adjunct in similar presentations.

There are several documented surgical techniques for treatment of aneurysms of the hand. These include excision and ligation, end-to-end microsurgical anastomosis, vein grafting and arterial grafting. Excision and ligation is the most commonly-used technique.^4^ However, on-table testing suggestive of insufficient collateral supply necessitates grafting or direct anastomosis.

The fact that the patient did not have a history of trauma raises the question of whether repetitive vibrational trauma to the hands (e.g. as a result of playing instruments) can cause a true digital artery aneurysm. Or perhaps the combination of repetitive trauma with increased intraluminal pressure due to digital artery vasospasm in primary Raynaud Syndrome is implicated. The role of repeated trauma from holding a golf club may also be an influencing factor, particularly as this is an area that has significant contact with the golf club handle during ball strike.

This case report adds to the current sparse literature on digital artery aneurysms and their presentation, investigation and management. It demonstrates that magnetic resonance angiography is a useful imaging tool for preoperative planning, allowing for visualisation of collateral blood supply to areas distal to the aneurysm. It also supports what previous studies have demonstrated – that excision and ligation is a safe surgical option provided there is sufficient collateral circulation.
